# Constituents of the Fruits of *Citrus medica* L. var. *sarcodactylis* and the Effect of 6,7-Dimethoxy-coumarin on Superoxide Anion Formation and Elastase Release

**DOI:** 10.3390/molecules22091454

**Published:** 2017-09-01

**Authors:** Yu-Yi Chan, Tsong-Long Hwang, Ping-Chung Kuo, Hsin-Yi Hung, Tian-Shung Wu

**Affiliations:** 1 Department of Biotechnology, Southern Taiwan University of Science and Technology, Tainan 71005, Taiwan; yuyichan@stust.edu.tw; 2 Graduate Institute of Natural Products, College of Medicine, Chang Gung University, Taoyuan 333, Taiwan; htl@mail.cgu.edu.tw; 3 Research Center for Chinese Herbal Medicine, Research Center for Food and Cosmetic Safety, and Graduate Institute of Health Industry Technology, College of Human Ecology, Chang Gung University of Science and Technology, Taoyuan 333, Taiwan; 4 Department of Anesthesiology, Chang Gung Memorial Hospital, Taoyuan 333, Taiwan; 5 School of Pharmacy, College of Medicine, National Cheng Kung University, Tainan 701, Taiwan; z10502016@email.ncku.edu.tw (P.-C.K.); z10308005@email.ncku.edu.tw (H.-Y.H.); 6 Department of Pharmacy, College of Pharmacy and Health Care, Tajen University, Pingtung 907, Taiwan

**Keywords:** *Citrus medica* L. var. *sarcodactylis* Swingle, Rutaceae, sesquiterpene, citrumedin-C, superoxide anion formation, elastase release

## Abstract

Investigation of the chemical constituents from the fruits of *Citrus medica* L. var. *sarcodactylis* Swingle has led to the characterization of a new sesquiterpene **1** along with thirty-two known compounds. The structure of **1** was established on the basis of 2D NMR spectroscopic and mass spectrometric analyses, and the known compounds were identified by comparison of their physical and spectroscopic data with those reported in the literature. In addition, most of the isolated compounds were evaluated for the activity assayed by the in vitro inhibition of superoxide anion generation and elastase release by human neutrophils. The results showed that only 6,7-dimethoxycoumarin (**5**) exhibited significant inhibition of superoxide anion generation, with IC_50_ value of 3.8 ± 1.4 μM.

## 1. Introduction

**Figure 1:**
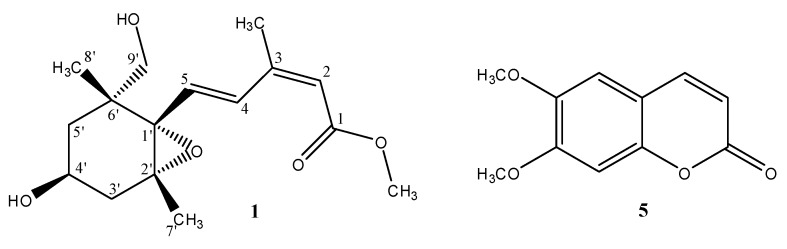
Structures of compounds **1** (relative configuration) and **5**.

*Citrus medica* L. var. *sarcodactylis* Swingle belongs to the genus *Citrus* (Rutaceae), and is commonly distributed in South and Southeast Asia. The plant is cultivated in the tropics and sub-tropics, such as India, Sri Lanka, Thailand, Vietnam, China, Japan, and Taiwan. Various species of *Citrus* have been used as foods, and their fruits, leaves, and roots have also been used as folk medicine or spice in Taiwan [1]. *Citrus* is one genus of the economically and medicinally important Rutaceae family, which has shown extensive biological and pharmacological activities, including antitumor [2,3,4,5,6,7], antiallergic [8], antioxidant [9,10,11,12], antiplatelet aggregation [13], anti-microbial [14,15,16,17], and anti-inflammatory activity [18,19,20,21,22]. Moreover, *Citrus* species have been reported to contain various bioactive coumarins, flavonoids, tetranortriterpenoids, monoterpenoids, and acridone alkaloids [23,24,25,26,27,28,29,30,31,32,33,34]. Previously, several anti-inflammatory compounds have been isolated from the stems and root barks of the titled plant [18]. In order to investigate the bioactive constituents from different parts of *C. medica* var. *sarcodactylis*, the fruits of this plant were selected for investigation. As a result, a new sesquiterpene **1** (Figure 1) and thirty-two known compounds have been characterized in the present work. The structural elucidation of **1** is described herein.

## 2. Results and Discussion

### 2.1. Purification and Characterization

The fresh fruits of *C. medica* var. *sarcodactylis* were pulverized into powder and extracted five times with methanol under reflux, and the combined extracts were concentrated to give a deep brown syrup. The crude extract was suspended in water and partitioned with CHCl_3_ to afford CHCl_3_ layer and water-soluble layer, respectively. Each layer was subjected to purification by a combination of conventional chromatographic techniques to result in one new compound (**1**). In addition, thirty-two known compounds were identified to be 5,7-dimethoxycoumarin (**2**) [3], xanthyletin (**3**) [18], oxypeucedanin hydrate (**4**) [35], 6,7-dimethoxycoumarin (**5**) [18], skimmin (**6**) [36], haploperoside A (**7**) [37], leptodactylone (**8**) [18], 7-methoxycoumarin (**9**) [18], scopoletin (**10**) [36], *cis*-head-to-tail-limittin dimer (**11**) [38], umbelliferone (**12**) [36], nordentatin (**13**) [18], limonin (**14**) [18], nomilin (**15**) [18], citrusin (**16**) [3], obacunone (**17**) [39], 3-(2-*O*-β-d-glucopyranosyl-4-methoxyphenyl)-propanoic acid (**18**) [40], *cis*-*p*-coumaric acid (**19**) [18], methyl vanillate (**20**) [41], methyl benzoate (**21**) [42], methyl paraben (**22**) [43], 4-hydroxy-phenethyl alcohol (**23**) [44], methyl-4-hydroxycinnamate (**24**) [43], coniferin (**25**) [45], syringin (**26**) [45], (*E*)-6-hydroxy-2,6-dimethylocta-2,7-dienoic acid (**27**) [46], a mixture of stigmasterol (**28**) [3] and β-sitosterol (**29**) [3], β-sitosteryl glucoside (**30**) [47], chrysoeriol 8-*C*-glucoside (**31**) [48], 1,2,3,4-tetrahydro-β-carboline-3-carboxylic acid (**32**) [49], and citrylidene malonic acid (**33**) [50], respectively. The structures of these compounds were identified by comparison of their physical and spectroscopic data with the values reported in the literature.

### 2.2. Structural Elucidation of Compound ***1***

**Figure 2:**
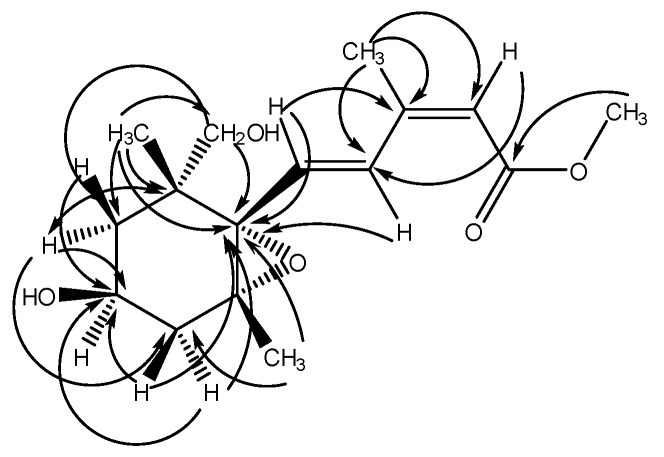
HMBC Correlations of **1**.

**Figure 3:**
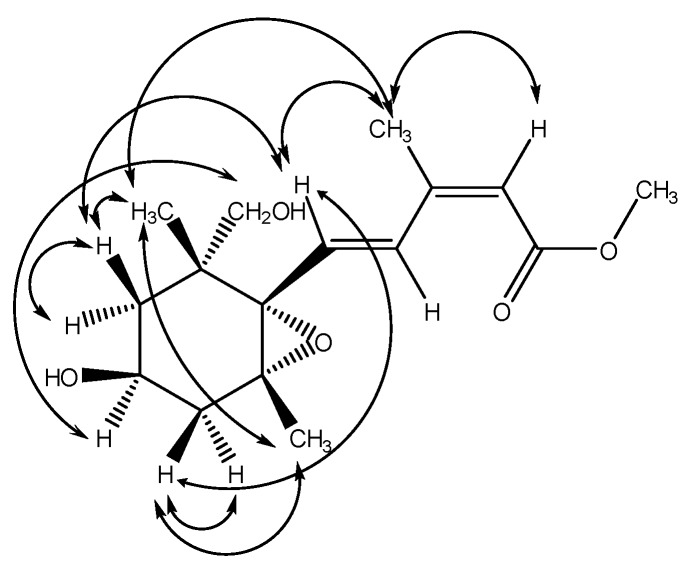
NOESY Correlations of **1**.

Citrumedin-C (**1**) was isolated as an optically active colorless powder. The HR-EI-MS analysis of **1** showed a molecular ion peak ([M]^+^) at *m*/*z* 296.1623, which was in agreement with the molecular formula C_16_H_24_O_5_. The UV spectrum appeared to show the maximum absorption at 266 nm. The IR spectrum revealed the presence of hydroxyl (3399 cm^−1^) and carbonyl groups (1701 cm^−1^). The ^1^H-NMR spectrum of **1** exhibited signals for one vinyl proton at δ 5.79 (1H, s), two *trans* olefinic protons at δ 8.00 (1H, d, *J* = 15.7 Hz) and 6.55 (1H, d, *J* = 15.7 Hz), two methyl singlets at δ 1.16 (3H) and 0.94 (3H), one methyl group attached to a double bond at δ 2.10 (3H, s), one methoxy group at δ 3.70 (3H, s), one oxymethine at δ 4.12 (1H, m), two oxymethylene protons at δ 3.82 (1H, d, *J* = 7.5 Hz) and 3.72 (1H, d, *J* = 7.5 Hz), one geminally coupled methylene at δ 2.03 (1H, dd, *J* = 13.7, 7.0 Hz) and 1.73 (1H, dd, *J* = 13.7, 10.4 Hz), and one methylene group attached with a methine at δ 1.86 (1H, dd, *J* = 13.5, 6.9 Hz) and 1.68 (1H, dd, *J* = 13.5, 13.5 Hz), respectively. The ^13^C-NMR spectrum exhibited a carbonyl signal at δ 168.2 (s); four olefinic carbons at δ 152.0 (s), 135.7 (d), 131.7 (d), and 118.3 (d); and two oxygenated carbons at δ 87.8 (s) and δ 83.3 (s) (Table 1), and these results were confirmed by the HSQC analysis. The COSY correlations of H-4′ (δ 4.12) to H-3′ (δ 2.03 and 1.73) and H-5′ (δ 1.86 and 1.68) suggested the presence of the partial structure (-CH_2_-CH(-O-)-CH_2_-). The six-membered C-ring was established by the HMBC correlations from H-5′ (δ 1.86) to C-6′ (δ 49.2) and C-1′ (δ 83.3), and from H-3′ (δ 2.03) to C-2′ (δ 87.8) and C-1′ (δ 83.3) (Table 1, Figure 2). The HMBC spectrum of **1** also showed the conjugated crosspeaks of H-5 (δ 6.55) to C-3 (δ 152.0)/C-1′ (δ 83.3), CH_3_-3 (δ 2.10) to C-2 (δ 118.3)/C-3 (δ 152.0)/C-4 (δ 131.7), and OCH_3_ (δ 3.70) to C-1 (δ 168.2), indicating that the partial structure (-CH=CH-C(-CH_3_)=CH-C(=O)-O-CH_3_) was substituted at C-1′. The HMBC correlations of H-8′ (δ 0.94) with C-6′ (δ 49.2)/C-5′ (δ 44.6)/C-1′ (δ 83.3)/C-9′ (δ 77.3), and of H-9′ (δ 3.72) with C-5′ (δ 44.6)/C-1′ (δ 83.3) revealed that the quaternary C-6′ was substituted with both methyl and hydroxymethyl groups. In addition, the correlations of the oxygenated quaternary carbon C-2′ (δ 87.8) and C-1′ (δ 83.3) with H-7′ (δ 1.16) supported that the C-2′ was substituted with a methyl group. According to the chemical shifts and the degree of unsaturation, it also suggested the presence of an epoxide ring at C-1′ and C-2′, and a hydroxyl group connected at C-4′, and these were further confirmed with the 2D spectroscopic analytical data. Thus, the structure of **1** was similar to abscisic acid (ABA) and xanthoxin in the phytohormone, which played the key role in biotic and abiotic stress responses [51]. The relative stereochemistry was confirmed by a NOESY experiment, which showed correlations of CH_3_-8′/CH_3_-7′, H-4′/H-9′, H-3′ (δ 1.73)/H-5, and H-3′ (δ 1.73)/CH_3_-7′, respectively (Figure 3). Therefore, the side chain at C-1′, two methyl substituents at C-2′ and C-6′, and the hydroxyl group at C-4′ were all in *cis* configuration. Conclusively, the structure of citrumedin-C (**1**) was assigned as shown based on the above-mentioned observations (See Supplementary Materials).

### 2.3. The Inhibitory Activity of Superoxide Anion Generation and Elastase Release

Most of the isolated compounds (compounds **2**–**6**, **10**, **12**, **14**–**18**, and **31**) were examined for their inhibition of superoxide anion generation and elastase release by human neutrophils in response to *N*-formyl-l-methionyl-phenylalanine/cytochalasin B (fMLP/CB) [52,53]. Only compound **5** (Figure 1) displayed inhibition percentages greater than 50% at the test concentration of 10 µM, and in the concentration range used, this compound displayed inhibitory effects in a dose-dependent manner. Compound **5** displayed significant inhibition of superoxide anion generation with IC_50_ value of 3.8 ± 1.4 μM, compared to the reference compound LY294002 with IC_50_ values of 0.4 ± 0.02 μM [52] (Table 2).

## 3. Materials and Methods

### 3.1. General Information

Melting points were determined using an MP-S3 apparatus (Yanaco, Tokyo, Japan). Optical rotations were measured using a P-2000 digital polarimeter (JASCO, Tokyo, Japan). UV spectra were recorded at room temperature on a U-0080-D spectrophotometer (Hitachi, Tokyo, Japan). IR spectra were obtained with an FT-IR Spectrum RX I spectrophotometer (PerkinElmer, Waltham, MA, USA). The EI-MS and HR-EI-MS were obtained on a VG-70-250S mass spectrometer. The ^1^H- and ^13^C-NMR, DEPT, COSY, HMQC, NOESY, and HMBC experiments were recorded on a Bruker AMX-400 spectrometer. Standard pulse sequences and parameters were used for the NMR experiments, and all chemical shifts were reported in parts per million (ppm, δ). Column chromatography (CC) was performed on silica gel (70–230 mesh and 230–400 mesh, Merck, Darmstadt, Germany), Diaion HP-20 (Mitsubishi, Tokyo, Japan), and C 18 (Sigma-Aldrich, St. Louis, MO, USA) gels, respectively, and preparative TLC (thin-layer chromatography) was conducted on Merck precoated silica gel 60 F254 plates, using UV light to visualize the spots. All solvents of extraction and isolation were purchased from Merck (Darmstadt, Germany).

### 3.2. Materials

The fruits of *Citrus medica* L. var. *sarcodactylis* Swingle was collected from the markets of Hualien County, Taiwan in September 2002, and identified by Prof. Chang-Sheng Kuoh. A voucher specimen (TSWu 20020923) has been deposited in the Herbarium of National Cheng Kung University, Tainan, Taiwan.

### 3.3. Extraction and Isolation

The fresh whole fruits of *Citrus medica* L. var. *sarcodactylis* Swingle (7.97 kg) were pulverized into small pieces and extracted with methanol under reflux (40 L × 5 h × 5). The resulting solution was then filtered and concentrated in vacuo to yield a crude extract. The MeOH extract (696 g) was suspended in distilled water and successively partitioned with chloroform yielding a chloroform layer (26 g) and water-soluble layer (670 g). The chloroform layer (26 g) was chromatographed directly on silica gel and eluted with a gradient of *n*-hexane and ethyl acetate to afford eight fractions. Fraction 3 was rechromatographed on silica gel and eluted with solvent of *n*-hexane–acetone (25:1) to give a mixture of β-sitosterol (**28**) and stigmasterol (**29**, 227 mg, R_f_ = 0.6), and citrylidene malonic acid (**33**, 1 mg, R_f_ = 0.4). Purification of fraction 4 by column chromatography with silica gel was eluted by gradient solvent mixture of chloroform and ethyl acetate (20:1 to 1:1) to afford 7-methoxycoumarin (**9**, 5 mg, R_f_ = 0.8), nordentatin (**13**, 1 mg, R_f_ = 0.7), nomilin (**15**, 19 mg, R_f_ = 0.6), methyl vanillate (**20**, 1 mg, R_f_ = 0.5), 4-hydroxy-phenethyl alcohol (**23**, 1 mg, R_f_ = 0.3), and methyl-4-hydroxycinnamate (**24**, 2 mg, R_f_ = 0.2), respectively. Separation of fraction 5 by column chromatography with silica gel eluted by chloroform and methanol (20:1) solvent mixture yielded xanthyletin (**3**, 13 mg, R_f_ = 0.7), leptodactylone (**8**, 1 mg, R_f_ = 0.5), umbelliferone (**12**, 11 mg, R_f_ = 0.4), and methyl paraben (**22**, 1 mg, R_f_ = 0.3). Fraction 6 was purified by silica gel CC eluted by a gradient solvent mixture of chloroform and methanol (20:1 to 1:1) to afford 5,7-dimethoxycoumarin (**2**, 198 mg, R_f_ = 0.6), 6,7-dimethoxycoumarin (**5**, 24 mg, R_f_ = 0.6), scopoletin (**10**, 21 mg, R_f_ = 0.5), *cis*-head-to-tail-limittin dimer (**11**, 1 mg, R_f_ = 0.3), and limonin (**14**, 59 mg, R_f_ = 0.2). Fraction 7 underwent a series of chromatographic separations on silica gel using chloroform/ methanol (15:1) as eluent to afford oxypeucedanin hydrate (**4**, 12 mg, R_f_ = 0.8), citrusin (**16**, 15 mg, R_f_ = 0.6), obacunone (**17**, 16 mg, R_f_ = 0.5), and (*E*)-6-hydroxy-2,6-dimethylocta-2,7-dienoic acid (**27**, 1 mg, R_f_ = 0.3). Recrystallization of fraction 8 produced the solid β-sitosteryl glucoside (**30**, 23 mg).

The water layer was subjected directly to Diaion HP-20 column chromatography, eluted by water and gradient with methanol to give six fractions. Fraction 4 was chromatographed over Sephadex LH-20 eluted with gradient solvent mixture of water and methanol to give skimmin (**6**, 17 mg, R_f_ = 0.7), haploperoside A (**7**, 1 mg, R_f_ = 0.6), methyl benzoate (**21**, 1 mg, R_f_ = 0.5), coniferin (**25**, 27 mg, R_f_ = 0.3), and syringin (**26**, 7 mg, R_f_ = 0.2). Fraction 5 was chromatographed on silica gel and eluted with solvent mixture of chloroform and methanol (5:1) to afford 3-(2-*O*-β-d-glucopyranosyl-4-methoxyphenyl)propanoic acid (**18**, 11 mg, R_f_ = 0.5) and 1,2,3,4-tetrahydro-β-carboline-3- carboxylic acid (**32**, 3 mg, R_f_ = 0.3). Fraction 6 was rechromatographed on silica gel and eluted with mixture of chloroform and methanol (9:1) to give citrumedin-C (**1**, 1 mg, R_f_ = 0.7), *cis*-*p*-coumaric acid (**19**, 1 mg, R_f_ = 0.6), and chrysoeriol 8-*C*-glucoside (**31**, 8 mg, R_f_ = 0.2).

Citrumedin-C (**1**): colorless powder, mp 138–139 °C; [α]_d_ +50.5 (CHCl_3_, *c* = 0.07); UV λ_max_ (MeOH) nm (log ε) 266 (4.38); IR(KBr) ν_max_ cm^−1^ 3399, 2920, 2874, 1701, 1601, 1447, 1373, 1234, 1165; ^1^H- and ^13^C-NMR data, see Table 1. EI-MS *m*/*z* 296 ([M^+^], 5), 278 (14), 264 (7), 246 (13), 220 (12), 188 (22), 169 (13), 154 (37), 135 (29), 122 (100); HR-EI-MS *m*/*z* [M]^+^ (Calcd. for C_16_H_24_O_5_ 296.1624, Found 296.1623).

### 3.4. Bioactivity Examination

#### 3.4.1. Preparation of Human Neutrophils

A study involving human neutrophils was approved by the Institutional Review Board at Chang Gung Memorial Hospital, Taoyuan, Taiwan, and was conducted according to the Declaration of Helsinki (2013). The written informed consent was obtained from each healthy donor before blood was drawn. Blood was drawn from healthy human donors (20–30 years old) by venipuncture into heparin-coated vacutainer tubes, using a protocol approved by the Institutional Review Board at Chang Gung Memorial Hospital. Blood samples were mixed gently with an equal volume of 3% dextran solution. Neutrophils were isolated with a standard method of dextran sedimentation prior to centrifugation in a Ficoll Hypaque gradient and hypotonic lysis of erythrocytes. The leukocyte-rich plasma was collected after sedimentation of the red cells for 30 min at room temperature, and was transferred to 20 mL Ficoll solution (1.08 g/mL) and spun down at 400 g for 40 min at 20 °C. The granulocyte/erythrocyte pellets were resuspended in ice-cold 0.2% NaCl to lyse erythrocytes [52]. After 30 s, the same volume of 1.6% NaCl solution was added to reconstitute the isotonic condition. Purified neutrophils were pelleted and then resuspended in a calcium (Ca^2+^)-free Hank’s balanced salt solution (HBSS) buffer at pH 7.4, and were maintained at 4 °C before use.

#### 3.4.2. Inhibition of Superoxide Anion Generation

The assay of the generation of superoxide anion was based on the superoxide dismutase (SOD)-inhibitable reduction of ferricytochrome c [52,54]. In brief, after supplementation with 0.6 mg/mL ferricytochrome c and 1 mM Ca^2+^, neutrophils (6 × 10^5^ cells/mL) were equilibrated at 37 °C for 2 min and incubated with drugs or an equal volume of vehicle (0.1% DMSO, negative control) for 5 min. Cells were activated with 100 nM fMLP during the preincubation of 1 µg/mL cytochalasin B (fMLP/CB) for 3 min. Changes in the absorbance with a reduction in ferricytochrome c at 550 nm were continuously monitored in a double-beam, six-cell positioner spectrophotometer with constant stirring (Hitachi U-3010). Calculations were based on differences in the reactions with and without SOD (100 U/mL) divided by the extinction coefficient for the reduction of ferricytochrome c (ε = 21.1/mM/10 mm at the concentration of 1 mM in cuvette with 1-cm optical path length).

#### 3.4.3. Inhibition of Elastase Release

Elastase release was measured by degranulation of azurophilic granules as described previously [52,54]. Experiments were performed using MeO-Suc-Ala-Ala-Pro-Val-*p*-nitroanilide as the elastase substrate. Briefly, after supplementation with MeO-Suc-Ala-Ala-Pro-Val-*p*-nitroanilide (100 µM), neutrophils (6 × 10^5^ cells/mL) were equilibrated at 37 °C for 2 min and incubated with test compounds or an equal volume of vehicle (0.1% DMSO, negative control) for 5 min. Cells were activated by 100 nM fMLP and 0.5 µg/mL cytochalasin B, and changes in absorbance at 405 nm were continuously monitored to assay elastase release. The results were expressed as the percent of elastase release in the fMLP/CB-activated, drug-free control system.

#### 3.4.4. Statistical Analysis

Normal distribution with Shapiro–Wilk was performed. The results are expressed as the mean ± SD and analyzed by analysis of variance (ANOVA) with post-hoc Bonferroni multiple comparisons tests. Calculations of 50% inhibitory concentrations (IC_50_) were computer-assisted (PHARM/PCS v.4.2). Statistical comparisons were made between groups using the Student’s *t* test. Values of *p* less than 0.05 were considered to be statistically significant.

## Figures and Tables

**Table 1:** ^1^H- and ^13^C-NMR spectral data of **1** (CD_3_OD, 400 MHz).

Position	δ_H_ (mult., J in Hz)	δ_C_
1		168.2
2	5.79 (1H, s)	118.3
3		152.0
4	8.00 (1H, d, 15.7)	131.7
5	6.55 (1H, d, 15.7)	135.7
1′		83.3
2′		87.8
3′	2.03 (1H, dd, 13.7, 7.0) 1.73 (1H, dd, 13.7, 10.4)	46.0
4′	4.12 (1H, m)	66.0
5′	1.86 (1H, dd, 13.5, 6.9) 1.68 (1H, dd, 13.5, 13.5)	44.6
6′		49.2
7′	1.16 (3H, s)	19.6
8′	0.94 (3H, s)	16.4
9′	3.82 (1H, d, 7.5) 3.72 (1H, d, 7.5)	77.3
CH_3_-3	2.10 (3H, s,)	21.2
OCH_3_	3.70 (3H, s)	51.6

**Table 2:** Inhibitory effects of isolated compounds on superoxide anion generation and elastase release by human neutrophils in response to *N*-formyl-l-methionyl-phenylalanine/cytochalasin B (fMLP/CB).

Compound	IC_50_ (μM) ^a^	
	Superoxide Anion Generation	Elastase Release
5	3.8 ± 1.4 ***	>10
LY294002 ^b^	0.4 ± 0.02 ***	1.5 ± 0.3 ***

^a^ Concentration necessary for 50% inhibition (IC_50_). Results are presented as means ± SD (*n* = 3–4). *** *p* < 0.001 compared with the control (DMSO). ^b^ A phosphatidylinositol-3-kinase inhibitor was used as a positive control.

## Supplementary Materials

^1^H-, ^13^C-NMR and 2D spectroscopic data of **1** are available online.
