# Supplementary material for: Constituents of the Fruits of Citrus medica L. var. sarcodactylis and the Effect of 6,7-Dimethoxy-coumarin on Superoxide Anion Formation and Elastase Release
Source: Molecules. 2017 Sep 1;22(9):1454. doi: 10.3390/molecules22091454 (PMC6151612; doi:10.3390/molecules22091454)
Supplement: Supplementary file 1 [file molecules-22-01454-s001.pdf]

Supplementary Materials

Constituents of the Fruits of *Citrus medica* L. var. *sarcodactylis* and the Effect of 6,7-Dimethoxycoumarin on Superoxide Anion Formation and Elastase Release

Yu-Yi Chan [1], Tsong-Long Hwang [2,3,4], Ping-Chung Kuo [5], Hsin-Yi Hung [5] and Tian-Shung Wu [5,6,*]

[1] Department of Biotechnology, Southern Taiwan University of Science and Technology, Tainan 71005, Taiwan
[2] Graduate Institute of Natural Products, College of Medicine, Chang Gung University, Taoyuan 333, Taiwan
[3] Research Center for Chinese Herbal Medicine, Research Center for Food and Cosmetic Safety, and Graduate Institute of Health Industry Technology, College of Human Ecology, Chang Gung University of Science and Technology, Taoyuan 333, Taiwan
[4] Department of Anesthesiology, Chang Gung Memorial Hospital, Taoyuan 333, Taiwan
[5] School of Pharmacy, College of Medicine, National Cheng Kung University, Tainan 701, Taiwan
[6] Department of Pharmacy, College of Pharmacy and Health Care, Tajen University, Pingtung 907, Taiwan

Contents

Fig. S1. ^{1}H NMR spectrum of **1**

Fig. S2. ^{13}C NMR spectrum of **1**

Fig. S3. COSY spectrum of **1**

Fig. S4. NOESY spectrum of **1**

Fig. S5. HMQC spectrum of **1**

Fig. S6. HMBC spectrum of **1**

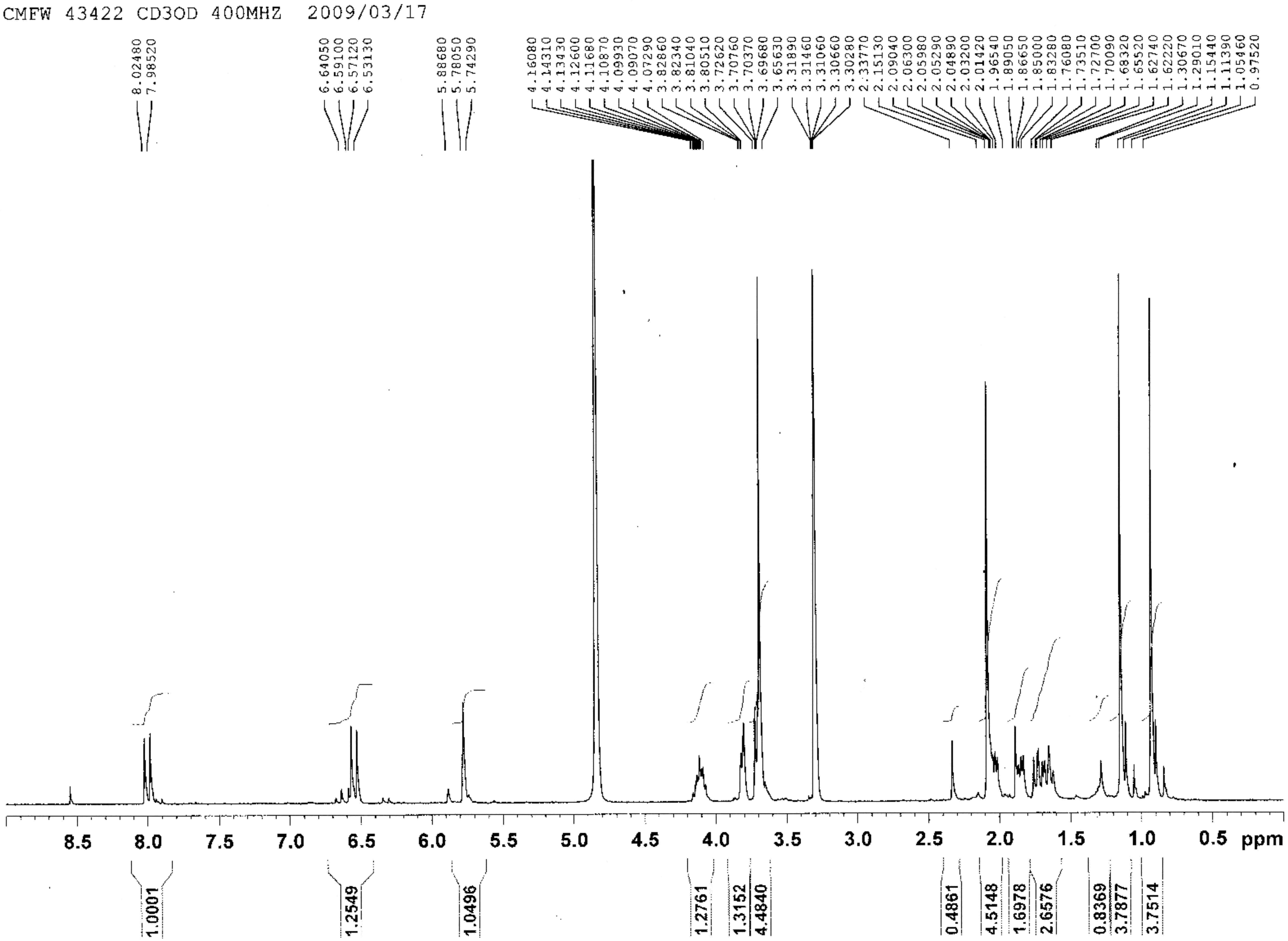
CMFW 43422 CD3OD 400MHZ 2009/03/17
8.5 8.0 7.5 7.0 6.5 6.0 5.5 5.0 4.5 4.0 3.5 3.0 2.5 2.0 1.5 1.0 0.5 ppm
1.0001
1.2549
1.0496
1.2761
1.3152
4.4840
0.4861
4.5148
1.6978
2.6576
0.8369
3.7877
3.7514

Fig. S1

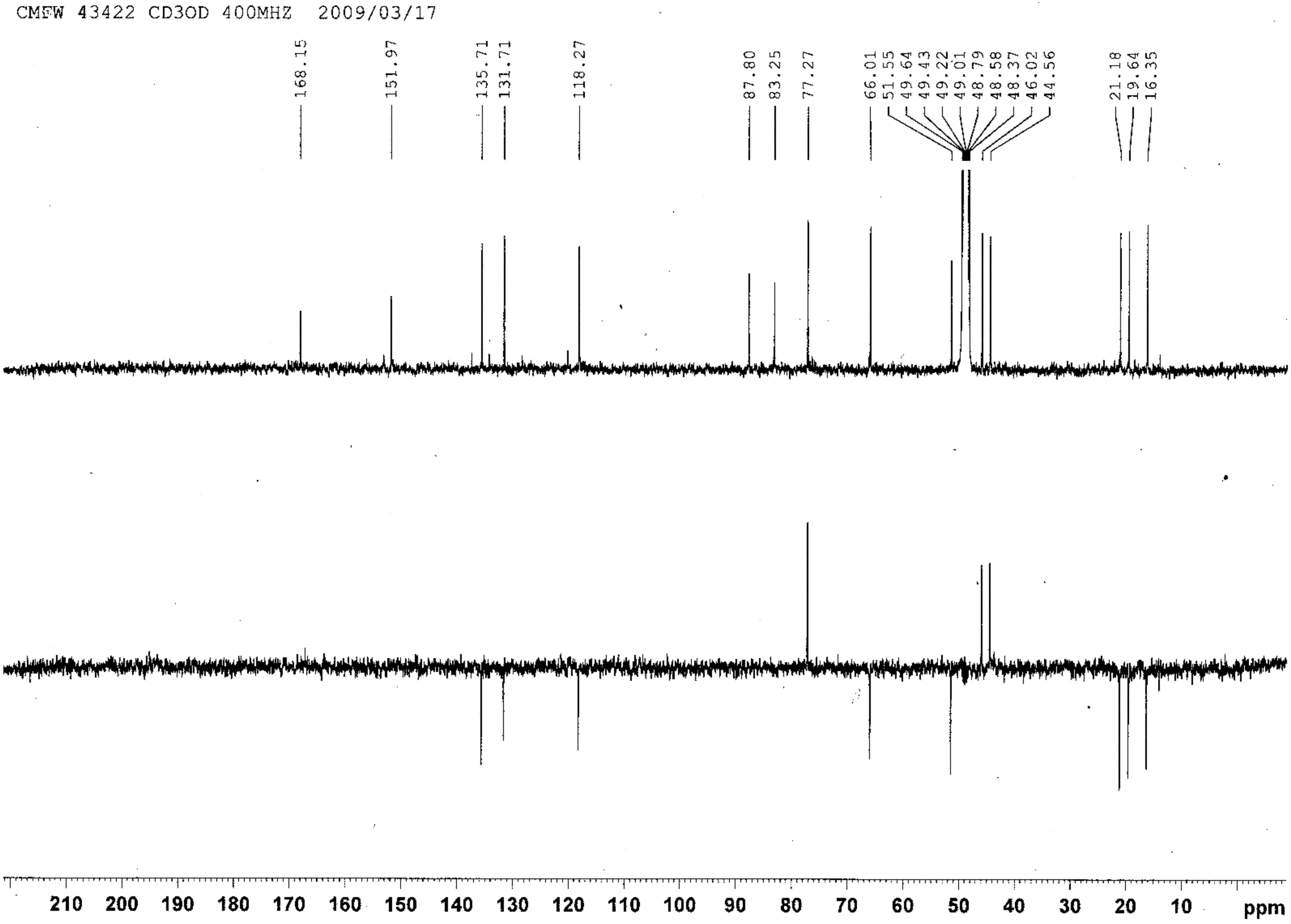
CMFW 43422 CD3OD 400MHZ 2009/03/17
168.15
151.97
135.71
131.71
118.27
87.80
83.25
77.27
66.01
51.55
49.64
49.43
49.22
49.01
48.79
48.58
48.37
46.02
44.56
21.18
19.64
16.35
210 200 190 180 170 160 150 140 130 120 110 100 90 80 70 60 50 40 30 20 10 ppm

Fig. S2

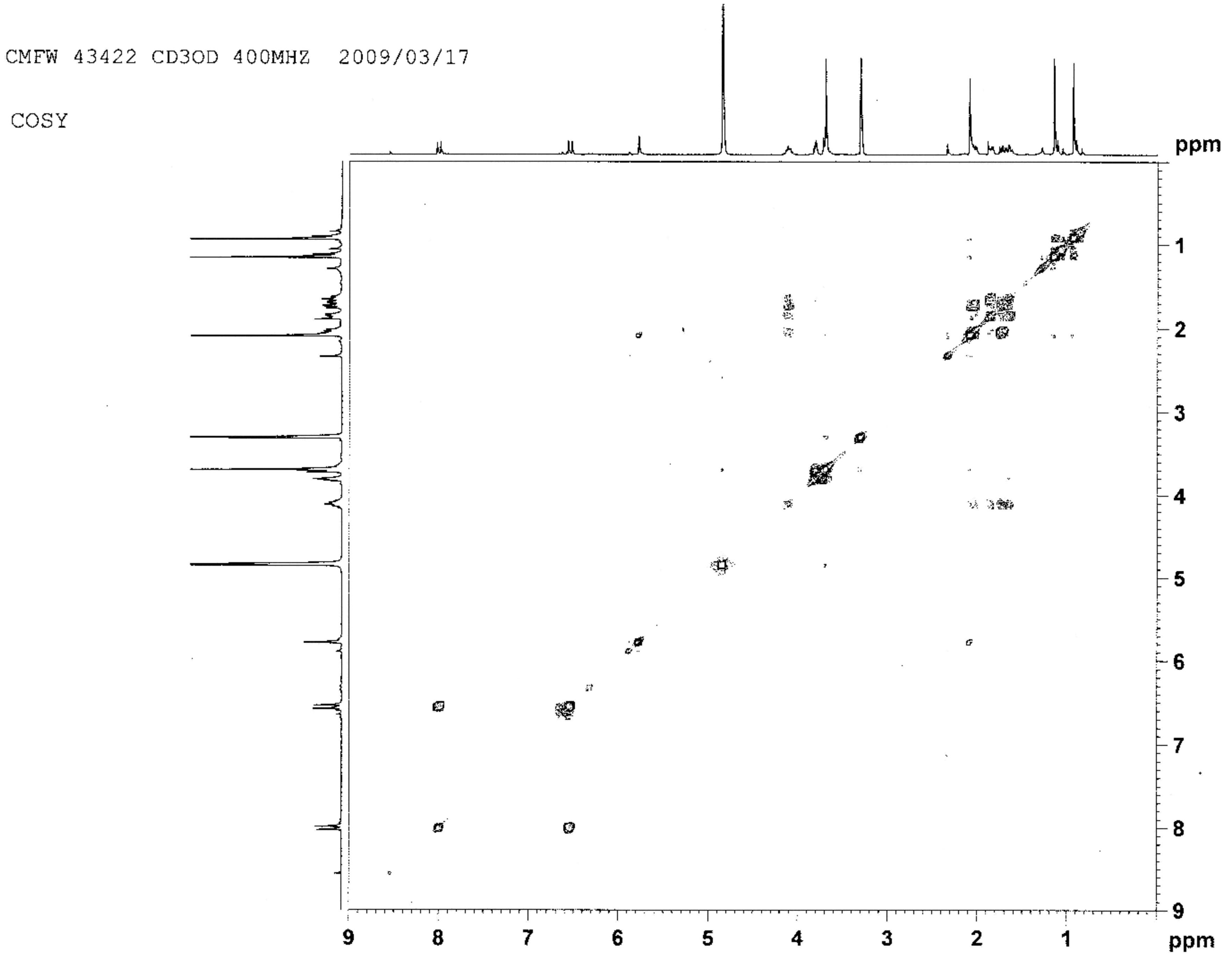
CMFW 43422 CD3OD 400MHZ 2009/03/17
COSY
ppm
1
2
3
4
5
6
7
8
9
9
8
7
6
5
4
3
2
1
ppm

Fig. S3

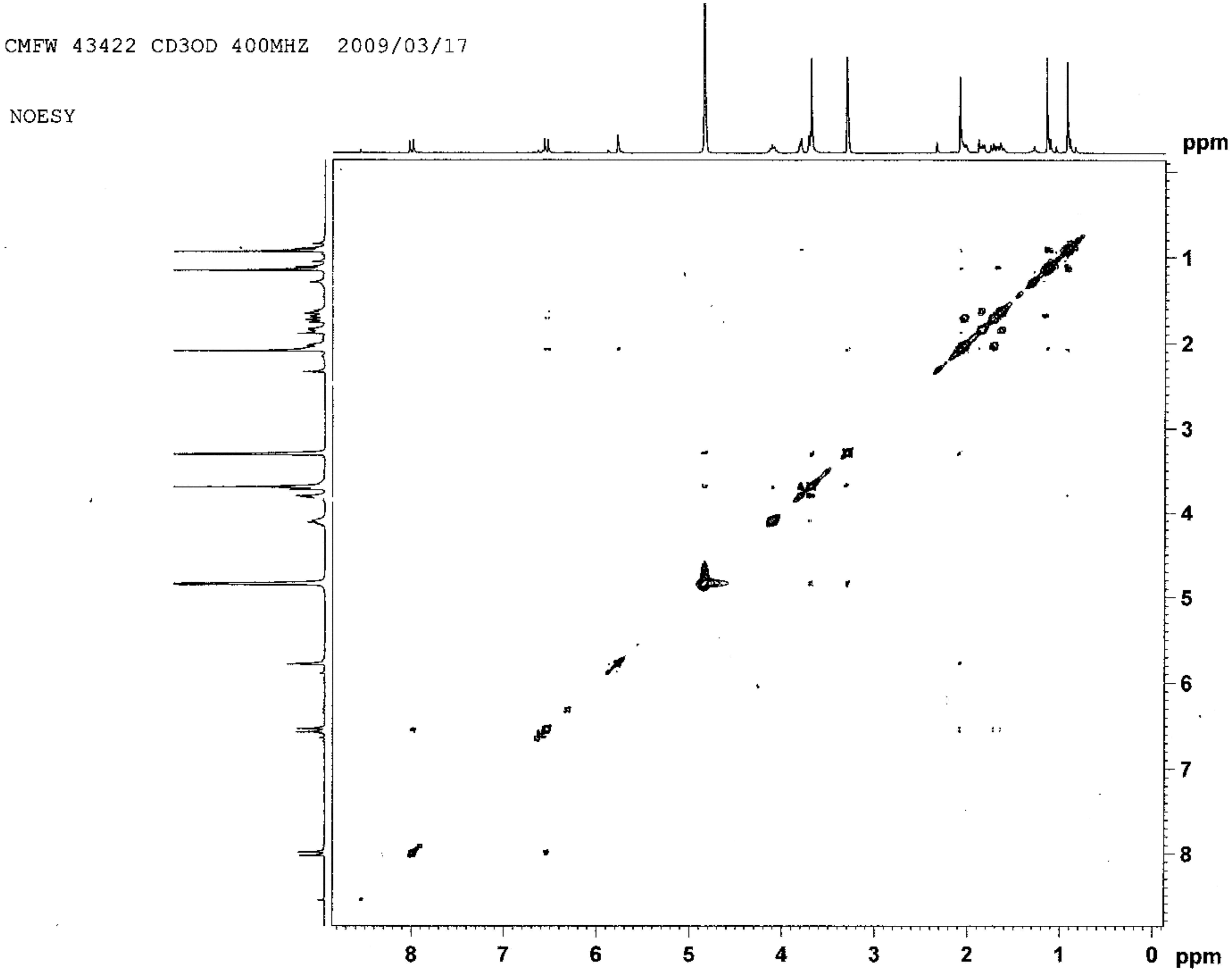

CMFW 43422 CD3OD 400MHZ 2009/03/17
NOESY
ppm
1
2
3
4
5
6
7
8
8 7 6 5 4 3 2 1 0 ppm

Fig. S4

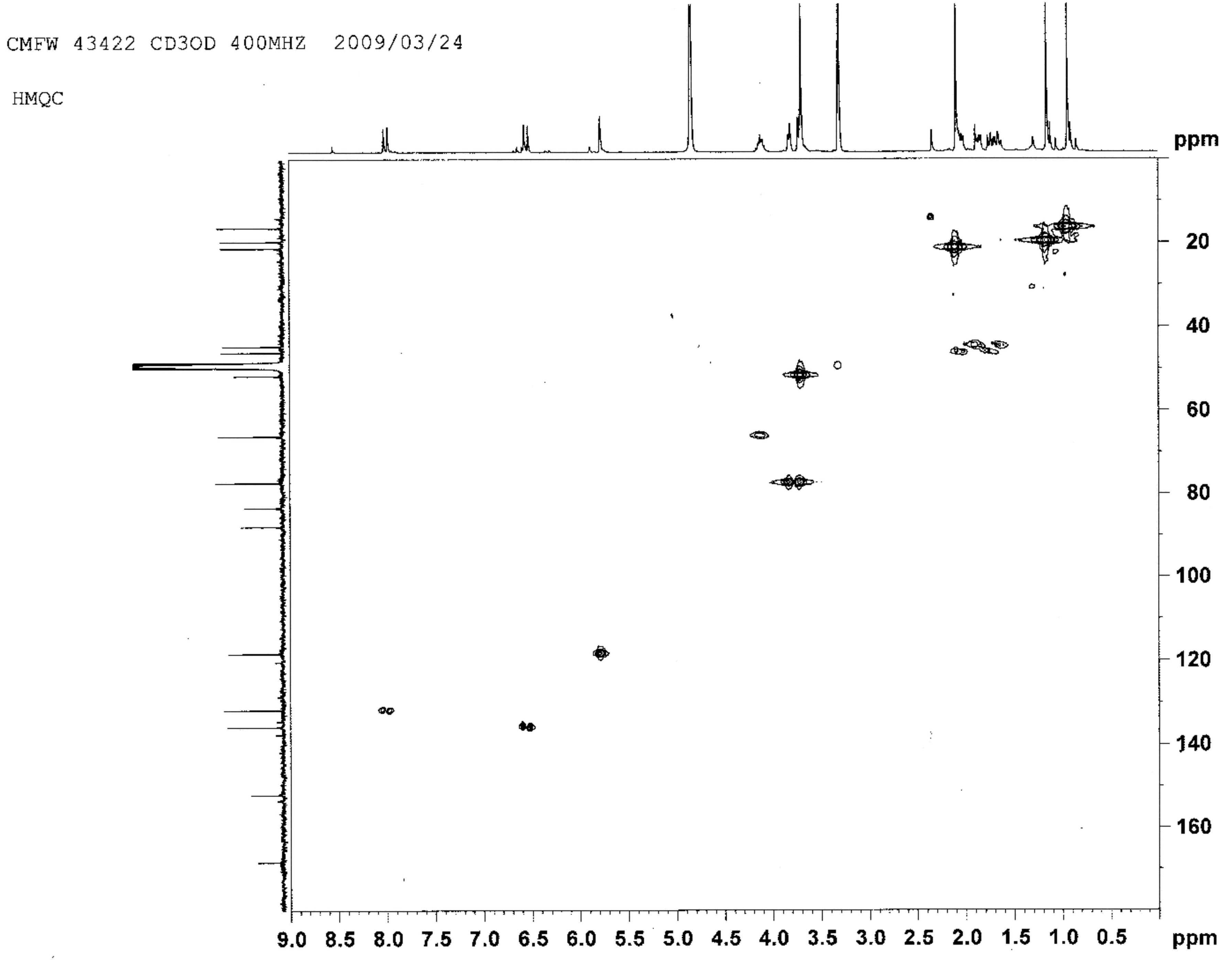
CMFW 43422 CD3OD 400MHZ 2009/03/24
HMQC
ppm
20
40
60
80
100
120
140
160
9.0 8.5 8.0 7.5 7.0 6.5 6.0 5.5 5.0 4.5 4.0 3.5 3.0 2.5 2.0 1.5 1.0 0.5
ppm

Fig. S5

CMFW 43422 CD3OD 400MHZ 2009/03/24

HMBC

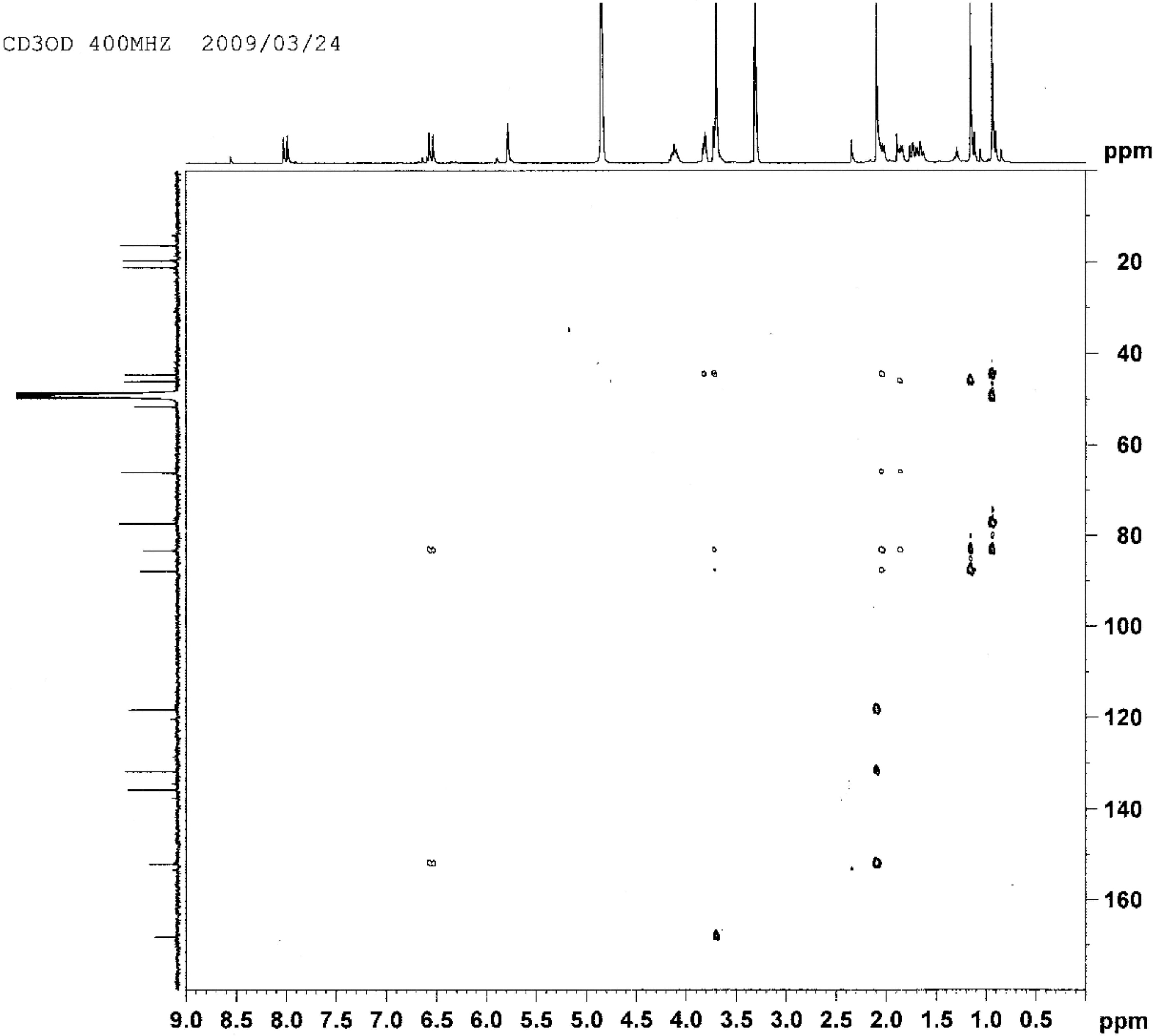

Fig. S6
